# Video Desnowing and Deraining via Saliency and Dual Adaptive Spatiotemporal Filtering

**DOI:** 10.3390/s21227610

**Published:** 2021-11-16

**Authors:** Yongji Li, Rui Wu, Zhenhong Jia, Jie Yang, Nikola Kasabov

**Affiliations:** 1 College of Information Science and Engineering, Xinjiang University, Urumqi 830046, China; liyongji@stu.xju.edu.cn (Y.L.); wurui@stu.xju.edu.cn (R.W.); 2 Institute of Image Processing and Pattern Recognition, Shanghai Jiao Tong University, Shanghai 200400, China; jieyang@sjtu.edu.cn; 3 Knowledge Engineering and Discovery Research Institute, Auckland University of Technology, Auckland 1020, New Zealand; nkasabov@aut.ac.nz

**Keywords:** video desnowing and deraining, saliency, adaptive filtering, outdoor vision sensing

## Abstract

Outdoor vision sensing systems often struggle with poor weather conditions, such as snow and rain, which poses a great challenge to existing video desnowing and deraining methods. In this paper, we propose a novel video desnowing and deraining model that utilizes the salience information of moving objects to address this problem. First, we remove the snow and rain from the video by low-rank tensor decomposition, which makes full use of the spatial location information and the correlation between the three channels of the color video. Second, because existing algorithms often regard sparse snowflakes and rain streaks as moving objects, this paper injects salience information into moving object detection, which reduces the false alarms and missed alarms of moving objects. At the same time, feature point matching is used to mine the redundant information of moving objects in continuous frames, and a dual adaptive minimum filtering algorithm in the spatiotemporal domain is proposed by us to remove snow and rain in front of moving objects. Both qualitative and quantitative experimental results show that the proposed algorithm is more competitive than other state-of-the-art snow and rain removal methods.

## 1. Introduction

Outdoor vision systems in traffic and safety applications have greatly promoted the development of society. Computer vision technologies, such as target tracking and human detection, are widely used. However, these technologies often confront challenges, such as heavy snow, rainstorms, strong winds and other poor weather conditions. Snowflakes and rain streaks can obscure key information in the video, and strong winds can shake the camera, which will make subsequent video processing more difficult. Therefore, removing snow and rain is an important part of computer vision.

In the early days, the photometric properties of rain were used to detect raindrops [1]. Some researchers utilized the direction and time attributes of rain streaks to remove rain [2,3]. However, the direction of snowfall is not consistent, so the direction attribute is not suitable for snow detection. Then, many researchers adopted filtering methods to remove snow and rain [2,4,5,6,7], but the cost of filtering is the loss of texture details in the background. Dictionary learning was adopted by some researchers to obtain rain dictionaries and non-rain dictionaries, but this method cannot completely remove rain [8].

Because the snowflakes and rain streaks in video do not cover the same pixels all the time, some researchers removed snow and rain [9,10,11] through the redundancy attributes between frames. However, the performance of this method is determined by selecting the number of frames and background pixels. Although this method can remove most snow and rain, it easily leaves holes and artifacts on moving objects.

Recently, a patch-based Gaussian mixture model was used to reconstruct a clear background [12], and the Markov random field (MRF) was used to detect moving objects in videos [12,13,14], but through these methods, the edges of moving objects are distorted. Low rank is an important attribute of snow and rain video. Some researchers use low-rank matrix decomposition to remove snow and rain [13,15]. This method can accurately restore the background. However, robust principal component analysis (RPCA), adopted by Tian et al. [15], and MRF, adopted by Ren et al. [13], often fail to detect small moving objects because these two methods have difficulty distinguishing between moving objects and sparse snowflakes, often removing moving objects as snowflakes, or retaining sparse snowflakes as moving objects. Although these methods [12,13,14,16] can deal with snow or rain videos containing moving objects, none of them can effectively remove snow or rain from moving objects.

To solve the above problems, this paper utilizes low-rank tensor decomposition to remove snow and rain in the video, which makes full use of the spatial location information and the correlation between the three channels of the color video. This decomposition is more robust to heavy snow and rainstorm videos. Conventional moving object detection methods [17,18] have difficulty in distinguishing between sparse snowflakes and moving objects without rich textures. In this paper, saliency detection and moving object detection are combined to extract moving objects separately [19,20,21] because rain streaks and snowflakes have no salience information in video.

To effectively remove snow and rain from moving objects, we utilize the sparsity of snowflakes and rain streaks to remove snow and rain in front of the moving object for the first time through the combination of feature point matching and adaptive minimum filtering in the time domain. To achieve the best effect from removing snow and rain on moving objects of different sizes, we utilize adaptive minimum filtering in the spatial domain to obtain the final moving objects without snow and rain.

The main contributions of this paper are as follows:

- Due to the interference of rain streaks and snowflakes, the existing snow or rain removal algorithms cannot effectively detect moving objects. We introduce a saliency map into moving object detection, which improves the ability of moving object detection in snow and rain videos because almost all moving objects in snow and rain videos have salience information, while snowflakes and rain streaks do not.
- Because snow and rain in videos cannot cover the same pixels all the time, feature point matching is utilized by us to address the time continuity of moving objects in snow or rain videos and mine the redundant information of moving objects in continuous frames. A dual adaptive minimum filtering method in the spatiotemporal domain is proposed by us to remove snow and rain in front of moving objects.
- In contrast to matrix decomposition, our tensor decomposition makes full use of the spatial location information and the correlation between the three channels of the color video. In our decomposition, the background is relatively static, and we uniformly regard sparse and dense snowflakes, rain streaks and moving objects as sparse components.

The rest of this paper is organized as follows: Section 2 systematically introduces the main related work in removing snow and rain. Section 3 describes the proposed method. Section 4 presents the experimental analysis and results. Section 5 discusses the advantages and disadvantages of our proposed method. Our summaries and prospects are arranged in Section 6.

## 2. Related Work

We give a review on the methods of video snow and rain removal. The methods of single image snow and rain removal are also introduced for literature comprehensiveness.

### 2.1. Video Snow and Rain Removal Methods

Early researchers utilized the physical properties of snow and rain and the time attributes of frames to remove snow and rain. Garg et al. [1] first discussed the photometric characteristics of raindrops and developed a rain detection method based on a linear spatiotemporal correlation model. Zhang et al. [2] introduced chromaticity and time attributes to the intensity fluctuation of rain pixels, and *k-*means clustering was utilized to distinguish background and rain in videos. However, these methods are not suitable for rain videos with moving objects.

Later, some researchers utilized filtering methods to remove snow and rain. Park et al. [4] adopted the Kalman filter to remove rain. Shen et al. [9] combined a saturation filter, difference filter and white filter to detect snow particles. However, these methods lose the texture details of the image.

The temporal correlation of frames was used by some researchers to remove snow and rain. Based on the frame difference method, Huiying et al. [10] added the constraints of area and bearing to improve the accuracy of snow detection. Yang et al. [11] combined the frame difference method and L0 gradient minimization to remove snow. Brewer et al. [3] proposed a method to distinguish between rain and moving objects based on the shape and angle of the rain streaks, but it is difficult to remove heavy rain with it. Barnum et al. [22] believed that snow and rain in videos obey blurred Gaussian distributions, but the robustness of this method is poor. Bossu et al. [23] detected snow and rain through selection rules based on photometry and size. However, when the directions of snow and rain are not consistent, the result is not ideal.

Recently, some researchers have considered the time correlation of snow and rain video frames. Kim et al. [16] took into account global motion, local motion and snowflakes of various sizes in their snow removal algorithm. First, snowflakes are detected by the correlation of frames, and then snowflakes and outliers are distinguished by sparse representation and support vector machine (SVM). Finally, low-rank matrix completion is utilized to reconstruct the video sequence. However, this method cannot effectively remove heavy snow because only the correlation of five frames is taken into account. Ren et al. [13] proposed an algorithm to remove snowflakes or rain streaks based on matrix decomposition, which distinguishes moving objects from sparse snowflakes by setting threshold values for the pixel intensity at specific locations in continuous frames. However, in some experiments, moving objects are often missed. Tian et al. [15] first obtained a clean background by global low-rank matrix decomposition. Then, block matching based on the average absolute difference and local low-rank decomposition were used to remove snow in front of moving objects. However, the complexity of this method is too high. It is difficult to extract the low-rank structure of moving objects, especially for nonrigid motion.

In addition, Islam et al. [24] proposed a hybrid technique, where physical features and data-driven features of rain are combined to remove rain streaks in videos. Jiang et al. [25,26] used the sparsity of rain streaks to remove rain in videos. Similarly, Li et al. [14] proposed online multiscale convolutional sparse coding (MS-CSC) to remove snow and rain and adopted the MRF to detect moving objects. An affine transformation operation was utilized to update the background. In contrast to the previous MS-CSC model [27] designed for rain removal in a prefixed length of video, this method adjusts the parameters according to the correlation between previous and current frames to cope with streaming videos with continuously increasing frames in real time. Wei et al. [12] proposed a patch-based Gaussian mixture model, which uses MRF to distinguish moving objects from rain. On this basis, Yi et al. [28] proposed an online patch-based Gaussian mixture rain removal model, which can learn parameters adaptively.

### 2.2. Single Image Snow and Rain Removal Methods

Many researchers are working on using a single image to remove snow and rain. To make the related work more comprehensive, we also introduce snow and rain removal methods for a single image. Guided filtering is the main method of rain and snow removal for a single image [5,6,7,29]. However, guided filtering loses the details of the image when removing snow and rain, which makes this method not suitable for images with rich textures. Wang et al. [8] proposed an image decomposition method based on dictionary learning and guided filtering to obtain a clean background by removing the imagery layer where the snow and rain components are located. Unfortunately, this method still blurs the texture details of the background.

Deep learning is widely used to remove snow and rain from a single image. Qian et al. [30] injected the attention mechanism into the generation and discrimination network to improve the rain removability of the network. However, this method may not always be effective in removing rain streaks in complex scenes. Ren et al. [31] utilized a multi-stream DenseNet to estimate the rain location map, a generative adversarial network to remove the rain streaks and a refinement network to refine the details. Chen et al. [32] proposed a snow removal algorithm based on the snow size and a transparency-aware filter consisting of a snow size recognizer and a snow removal system that can identify transparency. A transparency-aware module removes snow with different scales and transparency, and a modified partial convolution algorithm removes nontransparent snow. However, the background is easily distorted in the actual performance.

In addition, Jaw et al. [33] used a pyramidal hierarchical design with lateral connections across different resolutions. The high-level semantic features were combined with other feature maps at different scales to enrich the location information. Liu et al. [34] proposed a multistage snow removal network. The network is mainly composed of translucency recovery (TR) and residual generation (RG) modules. The former is used to restore the background obscured by translucent snow particles. The latter generates an area obscured by opaque snow particles via the unoccluded area and the recovered area of the former. Li et al. [35] designed a multiscale stacked densely connected convolutional network (MS-SDN) to detect and remove snow. The network consists of a multiscale convolution subnet for extracting feature maps and two stacked modified DenseNets for snow detection and removal.

The main differences between our approach and previous methods are as follows:

- The previous desnowing and deraining algorithm cannot distinguish between sparse snowflakes/rain streaks and moving objects in heavy snow/rainstorms. We utilize saliency map to guide moving object detection, which can effectively avoid the influence of snowflakes/rain streaks.
- The existing desnowing and deraining algorithms cannot effectively remove the snowflakes and rain streaks in front of the moving object. Additionally, some methods deform the moving object. To solve these problems, we combine feature point matching and dual adaptive spatiotemporal filtering, proposed by us, to remove snowflakes and rain streaks in front of moving objects.

## 3. Proposed Method

**Figure 1:**
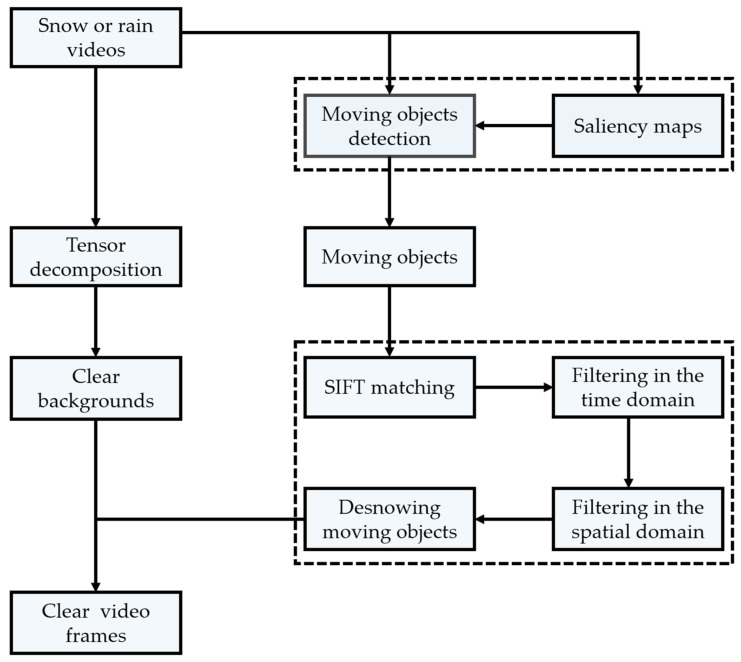
The flow diagram of our proposed algorithm.

In this section, we regard the snow video as a tensor, remove snow in the video by low-rank tensor decomposition, and then combine the saliency map with moving object detection to eliminate the interference of sparse snow, while extracting accurate moving objects. Finally, we utilize feature point matching and dual adaptive spatiotemporal filtering to remove the snow in front of the moving objects. The flow diagram of our proposed algorithm is shown in Figure 1.

### 3.1. Snow Video Background Modeling

The previous model converts snow video into the form of a matrix and then decomposes it into low-rank and sparse components. This decomposition can obtain a relatively clean background, but a major disadvantage of the matrix decomposition is that it can only deal with bidirectional (matrix) data, and the color snow/rain video data are a tensor.

The color frame is composed of three interrelated RGB channels. The matrix decomposition only deals with the three channels separately, which cannot make full use of the spatial location information, and the correlation between the three channels of the color video. This operation not only destroys the inherent structure of the original tensor, but also increases the computational cost of data analysis.

The natural advantage of the tensor is that one more dimension than the matrix can be used to store the RGB three-channel data. When decomposing the tensor, defining the tensor rank is an important problem. Unlike the rank of a matrix, researchers have many different definitions of the tensor rank, such as the CANDECOMP/PARAFAC (CP) rank [36], the Tucker rank [37], the tensor train (TT) rank [38], the tensor ring (TR) rank [39], and the tensor tubal rank [40]. In the restoration of color images and videos, the tensor tubal rank model based on the tensor–tensor product and tensor singular value decomposition (t-SVD) shows better performance than other rank models. The definitions of the tensor–tensor product, tensor singular value decomposition (t-SVD), tensor tubal rank, and tensor nuclear norm can be found in [41,42].

For a video sequence with a frame size of h×w and k frames, we consider a three-dimensional tensor $M\in {\mathbb{R}}^{n_{1}\times n_{2}\times n_{3}}$, where ℝ denotes the real number field. More precisely, the snow video is reshaped into a three-dimensional tensor 3×(hw)×k. Throughout this paper, we denote tensors by boldface Euler script letters. Our model is described as follows:

(1) $$\begin{array}{l} \underset{L,S}{\min}{\left\|L\right\|}_{*}+\lambda {\left\|S\right\|}_{1}\\ s.t.\;M=L+S,\; \end{array}$$

where M is the reshaped input video, $M\in {\mathbb{R}}^{3\times (hw)\times k}$, L is the low-rank background, S is the sparse component, ${\left\|\;•\;\right\|}_{*}$ denotes the nuclear norm, and ${\left\|\;•\;\right\|}_{1}$ denotes the $\ell_{1}-\mathrm{norm}$. Generally, $\lambda =1/\sqrt{(hw)k}$.

Then, the augmented Lagrangian function of (1) is as follows:

(2) $$L(L,\;S,\;\Lambda ,\;\beta )={\left\|L\right\|}_{*}+\lambda {\left\|S\right\|}_{1}+\langle \Lambda ,\;L+S-M\rangle +\frac{\beta }{2}{\left\|L+S-M\right\|}_{F}^{2},$$

where Λ is the Lagrange multiplier, β is the penalty parameter, 〈•〉 denotes the inner product, and ${\left\|\;•\;\right\|}_{F}^{2}$ denotes the square of the Frobenius norm.

We iteratively solve the optimization problem through the framework of the alternating direction method of multipliers (ADMM) algorithm:

(3) $$\left\{ \begin{array}{l} L_{k+1}=\underset{L}{\arg\min}{\left\|L\right\|}_{*}+\frac{\beta_{k}}{2}{\left\|L+S_{k}-M+\frac{\Lambda_{k}}{\beta_{k}}\right\|}_{F}^{2}\\ S_{k+1}=\underset{S}{\arg\min}\lambda {\left\|S\right\|}_{1}+\frac{\beta_{k}}{2}{\left\|L_{k+1}+S-M+\frac{\Lambda_{k}}{\beta_{k}}\right\|}_{F}^{2}\\ \Lambda_{k+1}=\Lambda_{k}+\beta_{k}(L_{k+1}+S_{k+1}-M)\\ \beta_{k+1}=\min(\beta_{k},\;\beta_{\max}) \end{array}\right.,$$

**Figure 2:**
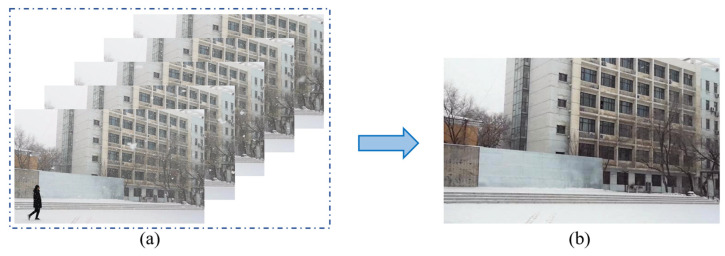
Extracting the low-rank background (**b**) from a snow video sequence (**a**).

The following equation serves as the stopping criterion for the above iterations:

(4) $$\min\left\{\begin{array}{l} {\left\|L_{k+1}+S_{k+1}-M\right\|}_{\infty },\\ {\left\|L_{k+1}-L_{k}\right\|}_{\infty },\\ {\left\|S_{k+1}-S_{k}\right\|}_{\infty } \end{array}\right\}\leq \varepsilon ,$$

where ε is a very small number, e.g., 1 × 10^−6^. Figure 2 shows the extracted low-rank component L of a snow video.

### 3.2. Moving Object Modeling

The conventional moving object detection methods have difficulty segmenting a complete moving object, and sparse snow is often recognized as a moving object, which results in snow that cannot be completely removed. To solve this problem, our proposed method combines the advantages of moving object detection and saliency detection, which introduces saliency items to form a new objective function. Specifically, we use a saliency map to guide moving object detection to strengthen the detectability of moving objects and weaken the impact of moving snow because snow tends to occupy most of the frame, which is not salient, while the moving object is salient. With the combination of a saliency map and the motion detection, a complete moving object can be extracted separately.

In snow videos, moving objects without rich texture are prone to not being detected. To reduce false alarms and missed alarms, a saliency map is incorporated into an incremental subspace analysis framework, more accurate moving objects can be extracted. Our objective function systematically takes into account the properties of sparsity, low rank, connectivity, and saliency. The imposed saliency map avoids the interference of snow, and the connectivity plays a smooth role in the moving objects.

In the snow video, $c\in {\mathbb{R}}^{N\times 1}$ denotes the current frame, where N is the number of pixels in the frame, i.e., N=h×w. The goal is to find the locations of the moving objects in the current image c. The moving object locations are represented by a foreground indicator vector $\bar{f}\in {\left\{0,1\right\}}^{N}$, where 0 denotes the background and 1 denotes the foreground. The negative of the background indicator vector $\bar{b}$ is identical to the foreground indicator vector $\bar{f}$, i.e., $\bar{f}=1-\bar{b}$, where $1\in {\mathbb{R}}^{N\times 1}$, and the elements are all 1. $\bar{b}$ is obtained by binarizing the background vector b.

The background vector is obtained by the following minimization problem:

(5) $$\underset{b,U,v}{\min}\sum_{i=1}^{N}\left[\begin{array}{l} \frac{1}{2}b_{i}{\left(U_{i}v-c_{i}\right)}^{2}+\\ \beta (1-b_{i})-\alpha b_{i}(1-s_{i}) \end{array}\right]+\lambda {\left\|Db\right\|}_{1},$$

where $U\in {\mathbb{R}}^{N\times m}$ is a subspace matrix whose columns are orthonormal, m is the number of columns of U, and $U_{i}$ stands for the ith row of U. The coefficient vector $v\in {\mathbb{R}}^{m\times 1}$ is the low-dimensional representation of frame c in the subspace spanned by the rows of U. $s\in {\mathbb{R}}^{N\times 1}$ is the saliency map obtained by some salient object detection algorithms, such as those in [43,44,45], and $s_{i}$ is the ith element of s. $D={\left[D_{h},D_{v}\right]}^{T}$ is a difference matrix, and $D_{h}$ and $D_{v}$ are forward finite-difference operators in the horizontal and vertical directions, respectively. α, β and λ are the balancing parameters.

In Equation (5), $U_{i}v$ is the reconstruction of the background, and $U_{i}v-c_{i}$ measures the similarity between $U_{i}v$ and $c_{i}$. The second term $\left(1-b_{i}\right)$ makes the estimated foreground much sparser to avoid the interference of snow. The connectivity term ${\left\|Db\right\|}_{1}$ is minimized to smooth the foreground and background. Minimizing the object saliency term $-b_{i}(1-s_{i})$ increases the chances that the foreground contains salient objects.

We utilize the alternating minimization method to seek the optimal variables b, U and v in turn. It is extremely difficult to seek the optimal solution of b directly. We let w=b and h=Dw. Equation (5) can be described as follows:

(6) $$\begin{array}{l} \underset{b,U,v}{\min}\sum_{i=1}^{N}\left[\frac{1}{2}b_{i}{\left(U_{i}v-c_{i}\right)}^{2}+\beta (1-b_{i})-\alpha b_{i}(1-s_{i})\right]+\lambda {\left\|h\right\|}_{1}\\ s.t.\;w=b,h=Dw, \end{array}$$

With the Lagrange multiplier, the constraint term in Equation (6) is converted into the following unconstrained form:

(7) $$\begin{array}{l} \underset{b,U,v,h,w}{\min}\sum_{i=1}^{N}\left[\frac{1}{2}b_{i}{\left(U_{i}v-c_{i}\right)}^{2}+\beta (1-b_{i})-\alpha b_{i}(1-s_{i})]\right]+\lambda {\left\|h\right\|}_{1}+\\ \frac{\mu }{2}{\left\|w-b\right\|}_{2}^{2}+x^{T}(w-b)+\frac{\mu }{2}{\left\|h-Dw\right\|}_{2}^{2}+y^{T}(h-Dw), \end{array}$$

where $\mu /2{\left\|w-b\right\|}_{2}^{2}$ and $x^{T}(w-b)$ are obtained by converting w=b into the unconstrained optimization function, and the vector x is the Lagrangian multiplier. Similarly, $\mu /2{\left\|h-Dw\right\|}_{2}^{2}$ and $y^{T}(h-Dw)$ are obtained by converting the constraint h=Dw into the unconstrained optimization function, and the vector y is the Lagrangian multiplier.

We solve the optimization problem (7) alternately to obtain the optimal variables.

We update b when U,v,h,w,x and y are fixed, as follows:

(8) $$b_{i}=\frac{\beta +\mu \omega_{i}+x_{i}-\frac{1}{2}{\left(U_{i}v-c_{i}\right)}^{2}+\alpha (1-s_{i})}{\mu },$$

We update h when b,U,v,w,x and y are fixed, as follows:

(9) $$h=\arg\underset{h}{\min}\frac{\lambda }{\mu }{\left\|h\right\|}_{1}+\frac{1}{2}{\left\|h-Dw+y/\mu \right\|}_{2}^{2},$$

The optimal solution is given by the following equation:

(10) $$h=S_{\lambda /\mu }(Dw-\frac{y}{\mu }),$$

We update w when b,U,v,h,x and y are fixed as follows:

(11) $$w=\arg\underset{w}{\min}\frac{\mu }{2}{\left\|w-b\right\|}_{2}^{2}+x^{T}(w-b)+\frac{\mu }{2}{\left\|h-Dw\right\|}_{2}^{2}+y^{T}(h-Dw),$$

Equation (11) is a quadratic function of w. Hence, the unique solution is the following:

(12) $$w={\left(I+D^{T}D\right)}^{-1}\left[D^{T}(h+\frac{y}{\mu })+b-\frac{x}{\mu }\right],$$

We update x and y when b,U,v,h and w are fixed as follows:

(13) $$\left\{ \begin{array}{l} x+\mu (w-b)\to x\\ y+\mu (h-Dw)\to y\\ d\mu \to \mu \end{array}\right.,$$

where d is a parameter and its empirical value is 1.25.

We update U when b,v,h,w,x and y are fixed as follows:

(14) $$\begin{array}{l} U=\arg\underset{U}{\min}{\sum_{i}\frac{1}{2}b_{i}(U_{i}v-c_{i})}^{2}\\ s.t.\;UU^{T}=I, \end{array}$$

where I is the identity matrix.

v is the low-dimensional representation of c, which is given by the following:

(15) $$v=U^{T}c$$

### 3.3. Feature Point Matching and Dual Adaptive Spatiotemporal Filtering

In the adjacent frames, the change of the moving object is very small, but the snow moves very fast, which makes the feature point matching accurately match the moving object.

**Figure 3:**
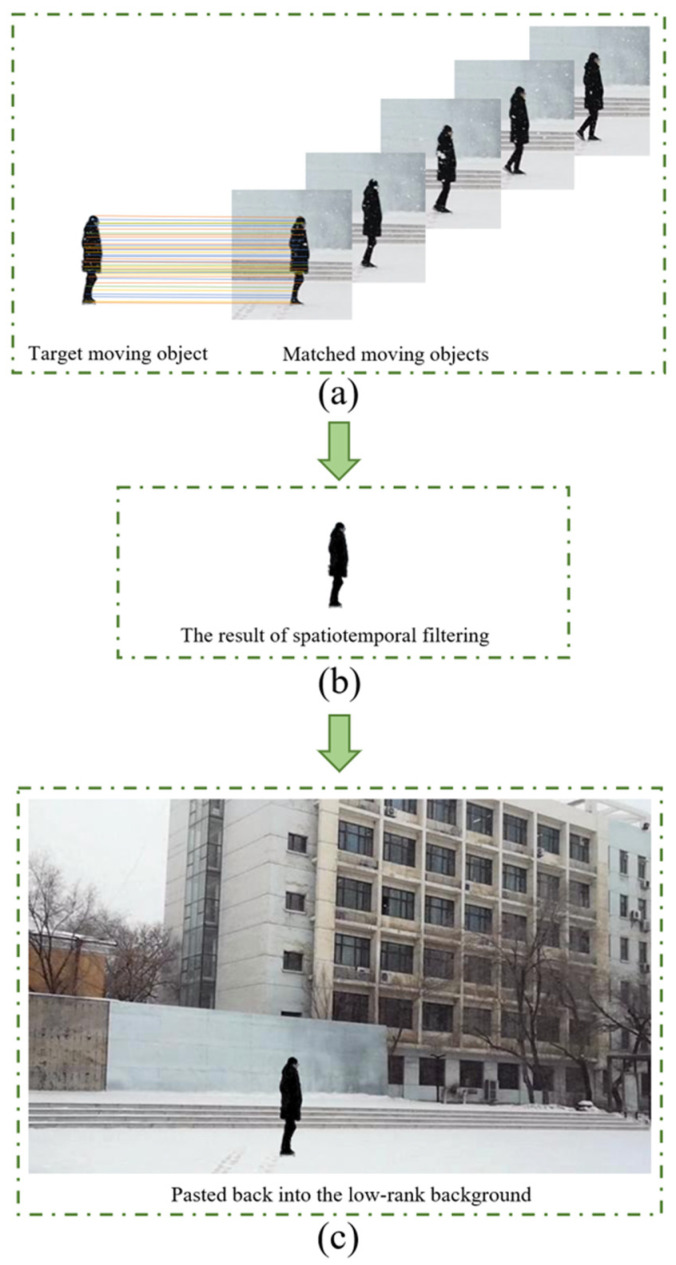
(**a**) The moving object matching processes, (**b**) the result of dual adaptive spatiotemporal filtering, (**c**) the clean video frame obtained by pasting the desnowing moving object back into a low-rank background.

We utilize the scale invariant feature transform (SIFT) matching method to match moving objects in snow videos. The SIFT matching algorithm is robust to changes in object translation, brightness and scale. It includes five steps: (1) We construct scale space and detect extreme points to obtain scale invariance. (2) Unstable feature points are filtered for accurate positioning. (3) We extract feature descriptors from feature points and assign direction values to feature points. (4) Feature descriptors are utilized to find matching points. (5) The Euclidean distance of the feature vector is used as a similarity measure of key points in two images. As shown in Figure 3, the SIFT matching we adopt can accurately match moving objects in different frames.

We paste the detected moving objects back into a low-rank background. When using feature point matching to remove snow in front of moving objects, one problem is that the number of matching frames directly determines the quality of snow removal in front of moving objects. To improve the robustness of the proposed method, we adaptively select the appropriate number of matching frames according to the speed of the moving object to strike a balance between over-smoothing and snow removal effects. General moving objects (such as pedestrians and cars) will produce unpleasant deformations in the spatiotemporal domain. If we measure the speed of the moving object according to the proportion of the coincident part of the moving object in the adjacent frame to the frame, there is a great error in the video with different resolutions. Therefore, we choose the proportion of the coincident part of the moving object in the adjacent frame to itself.

Each frame of the snow video reshapes a vector $c\in {\mathbb{R}}^{N\times 1}$. We set the pixel coincidence rate between the moving object in the target frame $c_{o}$ and the moving object in the previous frame $c_{o-1}$ to $\chi_{o-1}$. Similarly, the coincidence rate of the next frame is set to $\chi_{o+1}$. When the coincidence rate is less than 80%, subsequent frames are no longer matched:

(16) $$c_{o+i}=\left\{ \begin{array}{l} 0,\;\mathrm{if}\;\chi_{o+i}<80\%\\ 1,\;\mathrm{if}\;\chi_{o+i}\geq 80\% \end{array}\right.\;,\;i=\cdots ,-2,-1,\;1,\;2,\;\cdots$$

where 0 indicates that the current frame $c_{o}$ refuses to match $c_{o+i}$, 1 indicates that the current frame $c_{o}$ agrees to match $c_{o+i}$.

If there are E and F frames matching $c_{o}$ successfully forward and backward, respectively, then the reshaped matrix after matching is ${\mathbb{R}}^{N\times (E+F+1)}$. We select the smallest element value in each row as the result of time domain minimum filtering.

(17) $$c_{\tilde{o}}=\min[c_{o-E},\cdots ,c_{o-1},c_{o},c_{o+1},\cdots ,c_{o+F}],$$

where $c_{\tilde{o}}$ represents the result of adaptive minimum filtering in the time domain after the SIFT matching.

Because the time domain minimum filtering utilizes the correlation between frames, even if most of the moving object is covered by sparse snow, it can be recovered accurately.

In some cases, there are still unpleasant snow noises in the images after SIFT matching and minimum filtering in the time domain. To achieve a better snow removal effect, we introduce adaptive spatial domain minimum filtering:

(18) $${\tilde{H}}_{\left(i,j\right)}=\min\left\{\begin{array}{lllll} H_{\left(i-n,j-n\right)}, & \cdots , & H_{\left(i-n,j\right)}, & \cdots , & H_{\left(i-n,j+n\right)}\\ \vdots & & \vdots & & \vdots \\ H_{\left(i,j-n\right)}, & \cdots , & H_{\left(i,j\right)}, & \cdots , & H_{\left(i,j+n\right)}\\ \vdots & & \vdots & & \vdots \\ H_{\left(i+n,j-n\right)}, & \cdots , & H_{\left(i+n,j\right)}, & \cdots , & H_{\left(i+n,j+n\right)} \end{array}\right\},$$

where H represents the pixel on the moving object, and i and j represent the horizontal and vertical coordinates of the target pixel, respectively. $\tilde{H}$ is the result of spatial domain minimum filtering. The size of the sliding window depends on the size of the moving object.

## 4. Experiment

To show the superiority of our proposed method objectively and fairly, quantitative and qualitative evaluations are carried out in synthetic snow and rain videos, respectively. To further demonstrate the robustness of the proposed algorithm, the real snow and rain comparison scenes include heavy snow, rainstorms and dynamic background videos.

Our method is compared with state-of-the-art algorithms for removing snow and rain. The method of Kim et al. [16] was published in *Transactions on Image Processing* (TIP) in 2015 and not only effectively removes snow and rain, but it also has high robustness for dynamic scenes. The method of Wang et al. [8] was published in *Transactions on Image Processing* (TIP) in 2017, and it can remove snow and rain from a single image well. The algorithm of Li et al. [14] was published in *Transactions on Image Processing* (TIP) in 2021. Because this method updates parameters according to continuously increasing frames in real time, it can effectively remove snow and rain from dynamic scenes. The algorithm of Chen et al. [32] was presented at the *European Conference on Computer Vision* in 2020 and is currently the best snow removal method based on deep learning. All experiments were implemented on a PC with an i7 CPU and 32 GB RAM.

### 4.1. Comparation on Synthetic Snow and Rain Videos

We select two videos in CDNET database [46]. One of the scenes is called pedestrians, and the other is a challenging traffic intersection. Different degrees of snow and rain are added to the two videos. First, we qualitatively evaluate the snow and rain removal effect of the proposed algorithm and the four comparison algorithms. Then the quantitative evaluation results are given by comparing the peak signal-to-noise ratio (PSNR), the structural similarity (SSIM) [47], the feature similarity containing the chrominance information (FSIMc) [48] and the visual information fidelity (VIF) [49].

**Figure 4:**
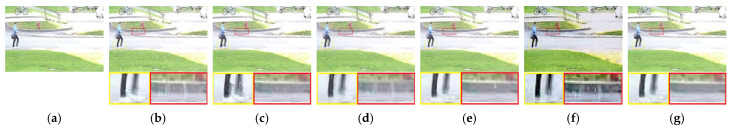
Comparison on a synthetic snow video. (**a**) Ground truth, (**b**) input, (**c**) Kim et al. [16], (**d**) Wang et al. [8], (**e**) Li et al. [14], (**f**) Chen et al. [32], (**g**) proposed method.

**Figure 5:**
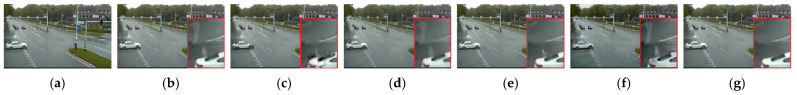
Comparison on a synthetic rain video. (**a**) Ground truth, (**b**) input, (**c**) Kim et al. [16], (**d**) Wang et al. [8], (**e**) Li et al. [14], (**f**) Chen et al. [32], (**g**) proposed method.

As shown in Figure 4, the method of Kim et al. [16] does not remove dense snowflakes and blurs the pedestrian’s legs. There is still much snow in the results of Wang et al. [8] and Chen et al. [32]. Among the four comparison methods, the performance of the method of Li et al. [14] is the best, but there is still snow in the result. There is little snow in our result. In Figure 5, the method of Kim et al. [16] still blurs the moving car. The methods of Wang et al. [8] and Chen et al. [32] do little to remove rain. Similar to the result in Figure 4, there is still a little rain left in the result of Li et al. [14]. Our snow removal effect is still the best.

To compare the snow removal effects of the five methods more objectively, Table 1 and Table 2 show the quantitative evaluation indices, such as PSNR, SSIM, FIMc and VIF, of each method. We calculate the average objective value of 200 frames of the above two videos. Our results are the best in every evaluation index, mainly because our proposed algorithm can effectively distinguish background from snow and rain. The method of Wang et al. [8] blurs the background, and the method of Chen et al. [32] distorts the background, which leads to the decline of their indices.

### 4.2. Comparation on Real Snow and Rain Videos

To further test the snow removability of our algorithm, in this section, we compare the proposed method with the four methods in real snow and rain videos.

**Figure 6:**
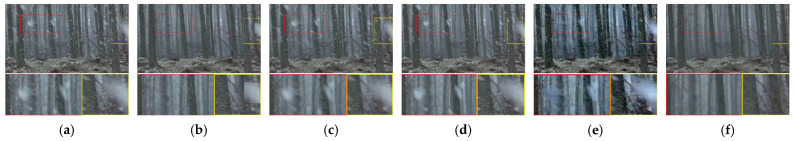
Comparison on a real snow video. (**a**) Input, (**b**) Kim et al. [16], (**c**) Wang et al. [8], (**d**) Li et al. [14], (**e**) Chen et al. [32], (**f**) proposed method.

**Figure 7:**
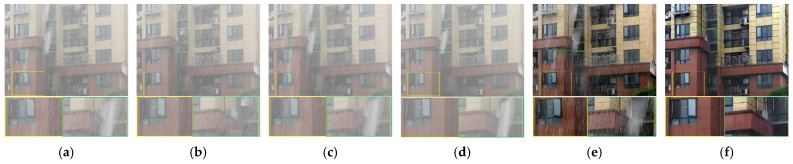
Comparison on a real rain video. (**a**) Input, (**b**) Kim et al. [16], (**c**) Wang et al. [8], (**d**) Li et al. [14], (**e**) Chen et al. [32], (**f**) proposed method.

Figure 6 shows a heavy snow scene, and Figure 7 shows a rainstorm scene. Although the methods of Li et al. [14] and Chen et al. [32] remove dense snow and rain, they cannot remove sparse snowflakes and rain streaks. The result of Kim et al. [16] is much better than the first two, but some snow and rain remain. Wang et al. [8] only removes dense snow and rain at the expense of image texture details. Because the main idea of this method is to remove snow and rain by filtering, the loss of background details is inevitable. In contrast, our method not only removes sparse and dense snow and rain, but it also restores a clear background.

**Figure 8:**
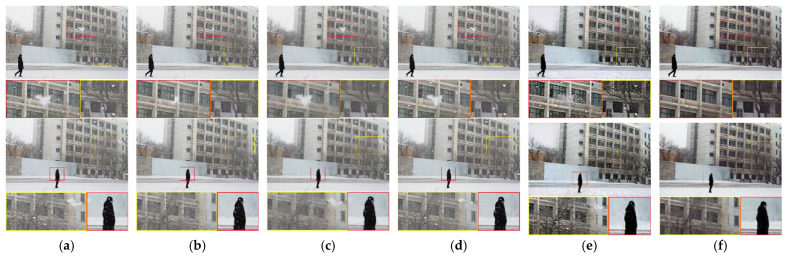
Comparison on a real snow video. (**a**) Input, (**b**) Kim et al. [16], (**c**) Wang et al. [8], (**d**) Li et al. [14], (**e**) Chen et al. [32], (**f**) proposed method.

Figure 8 is taken from the snow scene with a pedestrian passing by the static camera, and the snow in front of the black clothes is very obvious. None of the four comparison algorithms remove the snow in front of the background. The method of Kim et al. [16] removes almost all the snow in the background, but unfortunately, the snow in front of the moving object is not removed. The method of Chen et al. [32] can effectively remove snow in front of the moving object, but this method causes distortion of the ground and sky. Our method can truly restore the background and moving objects.

**Figure 9:**
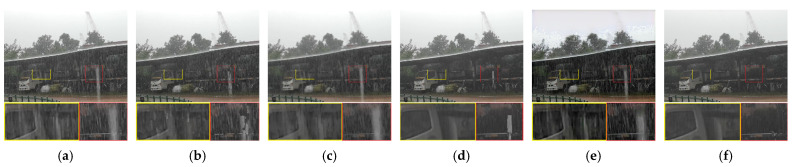
Comparison on a real rain video. (**a**) Input, (**b**) Kim et al. [16], (**c**) Wang et al. [8], (**d**) Li et al. [14], (**e**) Chen et al. [32], (**f**) proposed method.

Figure 9 shows a rainfall scene. The dark background highlights the bright rain streaks, which makes it more difficult to remove the rain. Because the rain streaks are very dense, the matrix completion of Kim et al. [16] cannot remove the dense rain streaks. The method of Li et al. [14] removes the dense rain streaks but does not completely remove the sparse rain streaks. The method of Wang et al. [8] limits the sparse rain streaks removability. Our proposed method removes almost all dense and sparse rain streaks.

**Figure 10:**
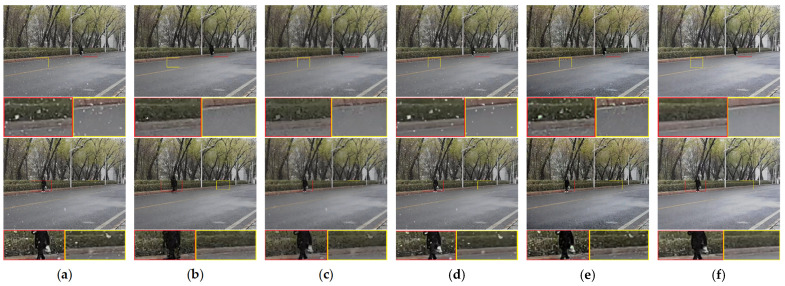
Comparison on a real snow video. (**a**) Input, (**b**) Kim et al. [16], (**c**) Wang et al. [8], (**d**) Li et al. [14], (**e**) Chen et al. [32], (**f**) proposed method.

Figure 10 is taken from the snow video containing swinging branches and a girl wearing a breathing mask. The slight swing of branches and the deformation of pedestrians pose a great challenge to snow removal. The method of Kim et al. [16] only uses the correlation between five frames to remove snow; the lack of the ability to identify moving objects leads to the wrong removal of the white bag, and the lack of an effective graph cut algorithm blurs the pedestrians. The methods of Wang et al. [8], Li et al. [14] and Chen et al. [32] cannot remove all of the snow in front of the pedestrian. In this scene, our results are still the best of the five methods.

**Figure 11:**
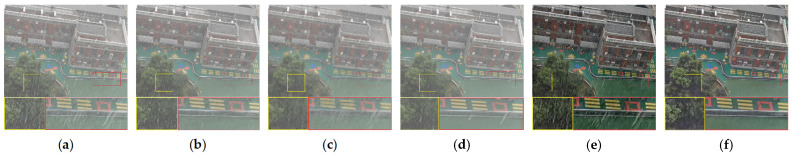
Comparison on a real rain video. (**a**) Input, (**b**) Kim et al. [16], (**c**) Wang et al. [8], (**d**) Li et al. [14], (**e**) Chen et al. [32], (**f**) proposed method.

Figure 11 is taken from a surveillance video. There are still some rain streaks left in the results of Kim et al. [16] and Li et al. [14]. The method of Wang et al. [8] seriously blurs the background because of its inherent limitations. Our method removes almost all of the rain streaks.

**Figure 12:**
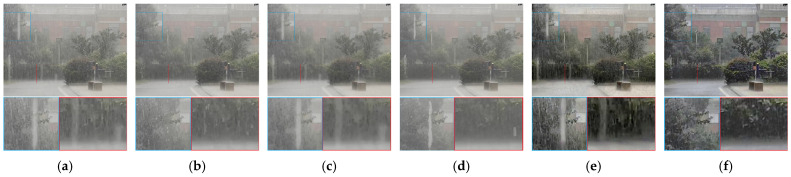
Comparison on a real rain video. (**a**) Input, (**b**) Kim et al. [16], (**c**) Wang et al. [8], (**d**) Li et al. [14], (**e**) Chen et al. [32], (**f**) proposed method.

The sparse rain streaks in Figure 12 pose a great challenge to the desnowing and deraining algorithms. The methods of Wang et al. [8], Li et al. [14] and Chen et al. [32] cannot remove the sparse rain streaks. The method of Kim et al. [16] limits its capability to address this continuous rain streaks. Comparatively, our proposed method still attains promising visual effect in rain removal.

### 4.3. Time Complexity Analysis

In this section, we discuss the runtime of our proposed algorithm and two video desnowing and deraining algorithms [14,16] for dealing with the synthetic snow video (Figure 4) and the real rain video (Figure 11). The resolution of the synthetic snow video is 360×240, and the resolution of the real rain video is 640×480. The number of frames in both videos is 100.

**Figure 13:**
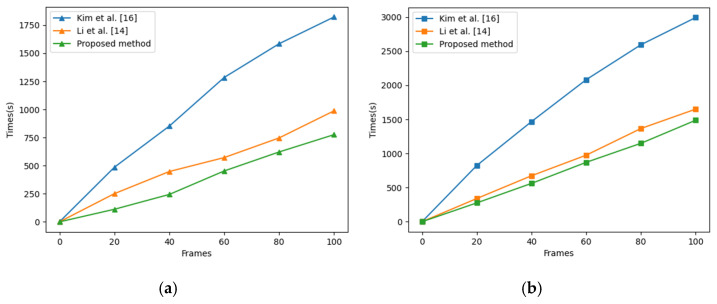
Runtime comparison of comparable methods on two videos. (**a**) The test object is the synthetic snow video (Figure 4). (**b**) The test object is the real rain video (Figure 11).

As can be seen from Figure 13, the method of Kim et al. [16] takes the longest time, mainly because it needs to calculate the snow or rain mask maps of each frame before removing the snow or rain. It takes about 50% to 70% of the whole time to calculate the snow or rain mask maps. The runtime of the method of Li et al. [14] is only lower than that of Kim et al. [16]; one of the main reasons is that it needs to learn parameters online. Whether the processing object is the real rain video or the synthetic snow video, our method is the most efficient.

## 5. Discussion

In contrast to the tensor decomposition in the literature [25], where the direction property of rain streaks is considered, because snowflakes do not have directional properties, our decomposition method uniformly regards sparse and dense snow and rain as sparse components when decomposing tensors. It can remove snowflakes and rain streaks at the same time since the snowflakes and rain streaks are always intrinsically sparser than the static and quasi-static backgrounds.

From the comparative experiments, it can be seen that the method of Wang et al. [8] is not suitable for snow samples with rich texture in the background. Regardless of how delicate the filtering is, the texture details of the background will be lost, and the dual adaptive spatiotemporal filtering proposed by us is no exception. The failure of Wang et al. [8] lies in global filtering. Unlike their method, our filtering works locally. Generally, the moving object occupies a very small part of the image, and the structure of the moving object is singular, which greatly avoids the loss of texture information caused by filtering.

We tested the performance of the method in over 30 different complex snowfall and rainfall scenes, including different light intensities and different intensities of snowfall and rainfall. The overall performance is good, but there are still three limitations. First, our algorithm works very well with videos taken by stationary or slow-moving cameras (such as surveillance), but it cannot address videos taken by fast moving cameras, due to the lack of video frame alignment technology. Second, when there are other saliency objects in the video background, the quality of the saliency image is reduced, and then the accuracy of moving object detection is affected. Furthermore, when the photometric similarity of moving objects and snowflakes is too high, snowflakes tend to be detected as moving objects. Third, if the moving object’s speed is too high, the SIFT matching may only match three to four frames, and the effect of the time domain minimum filtering is not good. In addition, too small moving objects may also lead to the failure of moving object detection. We will further endeavor on these degenerated cases for video snow and rain removal in our future research.

## 6. Conclusions

With the existing video snow and rain removal methods, it is difficult to meet the demands of outdoor vision sensing systems; one of the main reasons is that, using them, it is difficult to distinguish between sparse snowflakes/rain streaks and moving objects. To solve this problem, in this paper, we utilize tensor decomposition to remove sparse and dense snowflakes and rain streaks from the background, which makes good use of the spatial location information and the correlation between the three channels of the color video. Moving objects without rich texture information are easily confused with sparse snowflakes. By introducing salience information, the ability of moving object detection is improved. We use feature point matching to obtain the redundant information of the moving object between continuous frames, and then remove snow and rain in front of the moving object by the dual adaptive minimum filtering in the spatiotemporal domain. The experimental results show that our proposed method is superior to other state-of-the-art snow and rain removal methods.

In future research, we will seek more subtle saliency maps to further improve the ability to detect moving objects in snow and rain videos. The existing video snow and rain removal methods cannot effectively address snowfall and rainfall scenes with dynamic backgrounds. We will try to introduce video frame alignment technology [50] to address the snow and rain videos captured by mobile cameras.

## Figures and Tables

**Table 1:** Quantitative performance comparison of synthetic snow videos. All the results are the average of 200 frames.

Algorithm	Pedestrians			
	PSNR	SSIM	FSIMc	VIF
Kim et al. [16]	32.952	0.986	0.986	0.842
Wang et al. [8]	28.940	0.933	0.917	0.462
Li et al. [14]	35.395	0.987	0.988	0.832
Chen et al. [32]	25.963	0.898	0.916	0.506
proposed method	36.287	0.988	0.989	0.858

**Table 2:** Quantitative performance comparison of synthetic rain videos. All the results are the average of 200 frames.

Algorithm	twoPositionPTZCam			
	PSNR	SSIM	FSIMc	VIF
Kim et al. [16]	35.725	0.984	0.988	0.803
Wang et al. [8]	31.255	0.930	0.952	0.521
Li et al. [14]	37.848	0.982	0.984	0.795
Chen et al. [32]	23.698	0.837	0.895	0.501
proposed method	38.694	0.986	0.988	0.816

## Data Availability

We have evaluated our proposed method on publicly available datasets: the changedetection.net (CDnet) dataset. http://www.changedetection.net (accessed on 29 October 2021).
